# Modulation of incentivized dishonesty by disgust facial expressions

**DOI:** 10.3389/fnins.2015.00250

**Published:** 2015-07-21

**Authors:** Julian Lim, Paul M. Ho, O'Dhaniel A. Mullette-Gillman

**Affiliations:** ^1^Neuroscience and Behavioral Disorders Program, Duke-NUS Graduate Medical SchoolSingapore, Singapore; ^2^Singapore Institute for Neurotechnology, National University of SingaporeSingapore, Singapore; ^3^Department of Psychology, National University of SingaporeSingapore, Singapore

**Keywords:** decision making, moral, dishonesty, disgust, sensitivity, individual differences, social, cheating

## Abstract

Disgust modulates moral decisions involving harming others. We recently specified that this effect is bi-directionally modulated by individual sensitivity to disgust. Here, we show that this effect generalizes to the moral domain of honesty and extends to outcomes with real-world impact. We employed a dice-rolling task in which participants were incentivized to dishonestly report outcomes to increase their potential final monetary payoff. Disgust or control facial expressions were presented subliminally on each trial. Our results reveal that the disgust facial expressions altered honest reporting as a bi-directional function moderated by individual sensitivity. Combining these data with those from prior experiments revealed that the effect of disgust presentation on both harm judgments and honesty could be accounted for by the same bidirectional function, with no significant effect of domain. This clearly demonstrates that disgust facial expressions produce the same modulation of moral judgments across different moral foundations (harm and honesty). Our results suggest strong overlap in the cognitive/neural processes of moral judgments across moral foundations, and provide a framework for further studies to specify the integration of emotional information in moral decision making.

## Introduction

While dishonest Wall Street bankers and philandering politicians are roundly condemned in the court of public opinion, most people are not themselves perfectly honest. Small infractions, when multiplied over a population, can have a massive impact on society—for example, it is estimated that the US government has a half-trillion-dollar tax shortfall each year due to millions of US citizens under-reporting their income (Feige and Cebula, 2011). Considering its huge societal impact, dishonesty is an understudied phenomenon for which small interventions could produce large societal windfalls. Specifically, studying the modulation of moral judgment by social and emotional factors could point toward interventions to facilitate judgments across moral domains.

Making moral decisions involves the integration of multiple decision inputs, including utilitarian components (e.g., expected outcomes), deontological rules (e.g., “thou shalt not steal”), and socio-emotional factors. The socio-emotional factors have been the focus of much recent research (Greene et al., 2001; Wheatley and Haidt, 2005; Cummins and Cummins, 2012), and many investigators have tested the effects of disgust induction on moral judgments in particular due to their strong theoretical links (Rozin et al., 1999; Hutcherson and Gross, 2011). Early work suggested that disgust increases the severity of moral judgments by encouraging rule-based (deontological) over utilitarian thinking (Schnall et al., 2008a,b). However, a number of other studies have since contradicted these initial findings (La Rosa et al., 2012; Ugazio et al., 2012; Ong et al., 2014).

We recently specified that disgust primes modulate judgments of moral dilemmas based upon a third variable: individual sensitivity to disgust (Ong et al., 2014). In this series of experiments, we confirmed that disgust primes modulated the acceptability ratings of utilitarian actions. However, the direction and degree of that effect was moderated (*r* = 0.47) by each participant's sensitivity to disgust (Disgust-Scale Revised, Olatunji et al., 2007). Specifically, individuals with high disgust sensitivity found the utilitarian actions more acceptable, while low-sensitivity individuals found them less acceptable. This finding led us to suggest that the previous contradictory reports in the literature could be explained by this bi-directional function—as the identified modulation would be dependent on the mean disgust sensitivity of the investigated sample.

In our prior work, we used trolley-car, or sacrificial, dilemmas, in which participants judged the moral acceptability of an action that would hurt or kill one person to save multiple others (Foot, 1967; Unger, 1996; Greene et al., 2001). Such scenarios are helpful for initial exploration of moral decision-making processes; however, they may have low ecological validity as they are hypothetical and sometimes set in artificial or far-fetched contexts (Bauman et al., 2014; Kahane, 2015). This concern motivated us to probe the robustness of our found function using a non-hypothetical moral task. To achieve this, we employed a simple incentive-compatible monetary task that has been previously shown to elicit cheating behavior (Jiang, 2013; Ariely et al., 2014).

**Figure 1:**
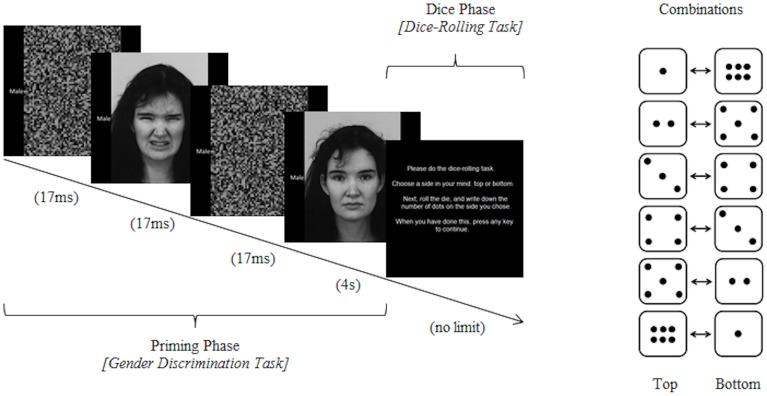
**Schematic of one trial of the task paradigm**. Subjects first performed the gender discrimination task, during which they were primed subliminally with a facial image with either a disgust or neutral expression. They then mentally selected “top” or “bottom” before rolling a physical die and writing down the outcome (which may not have been the true outcome of their decision).

For each trial of this task (Figure 1), subjects roll a six-sided die, from which they report the value of a pre-chosen side. Critically, they perform the task with little supervision and may easily write down each value dishonestly if they choose to do so. Further, the monetary payments participants received were dependent on the values that they reported, motivating them to cheat. To test the effect of our emotional modulation on this task, we primed subjects subliminally with a disgusted facial expression before half of the die-rolling trials, and compared the likely amount of dishonest reporting between this condition and the control of neutral facial expressions.

While it has been demonstrated that disgust can modulate the judgment of dishonest acts (Wheatley and Haidt, 2005), a recent fMRI study has also suggested that moral judgments of harm, dishonesty, and purity violations are subserved by distinct neural systems (Parkinson et al., 2011). This motivated us to test whether the bi-directional function we found using sacrificial dilemmas generalizes from the domain of harm to the domain of dishonesty. We tested the specific hypothesis that we would replicate our previous bi-directional function (Ong et al., 2014); that is, we predicted that priming with disgust facial expressions would modulate cheating behavior based on individual disgust sensitivity. In our previous experiment, individuals low on disgust sensitivity were less likely to endorse the acceptability of utilitarian actions, that is, relying more heavily on deontological rules. We predicted a parallel result in the honesty domain: that low sensitivity would lead to a decreased likelihood of cheating (i.e., a greater tendency to abide by the rules of the experiment) and high sensitivity reducing this likelihood.

## Materials and methods

### Participants

Participants were recruited via online advertisements on the National University of Singapore web portal and by word-of-mouth. Participants were screened to ensure that they had not previously participated in moral decision-making experiments conducted by our group. Fifty one participants (17 male) from ages 19 to 27 [mean = 21.6(2.15)] were recruited. Data from one participant was not recorded due to technical difficulties. Participants gave written consent before taking part in the study, and all tasks and procedures were approved by the Institutional Review Board of the National University of Singapore. A priori, we decided upon a sample size of 50 participants based on our found effects in the harm vignette studies (see Ong et al., 2014, which featured three samples of ~30 participants), and our desire to be able to identify potentially weaker effects.

### Procedure

Testing was conducted at the Centre for Life Sciences at the National University of Singapore. Each participant was run individually, with a single research assistant conducting all sessions. This research assistant was present in the room during the entire experiment, but was blind to the order of conditions for the participant. In order to mitigate experimenter-expectancy effects, verbal communication with the participant was minimized and the research assistant sat with his back to the participant during data collection.

Participants were first briefed on the gender discrimination and dice-rolling paradigms (see below), and practiced two trials with neutral priming. They were allowed to select the die they would use from a tray of a dozen identical dice. Importantly, they were then informed that their payment would be determined by random selection of one of the trials at the conclusion of the experiment, paid at a rate of 1 Singapore dollar (SGD) per pip.

Following the briefing and practice, subjects completed the Belief in Luck and Luckiness Scale (Thompson and Prendergast, 2013), administered to divert suspicion from our examination of cheating behavior. Subjects then performed two blocks of the dice-rolling paradigm, one primed with disgust facial expressions, and one primed with neutral facial expressions. Priming order was counterbalanced across subjects. Each block consisted of 20 trials of priming and dice-rolling. In between task blocks, subjects watched a 7-min video on coral reefs to minimize carry-over effects of the priming (same video clip as Ong et al., 2014).

At the conclusion of the two task blocks, subjects completed three further questionnaires: demographic data, the Duke University Religion Index (Koenig and Bussing, 2010) and the Disgust Sensitivity Scale-Revised (Olatunji et al., 2007). We then conducted a debriefing to check if subjects had detected the subliminal primes or guessed the purpose of the experiment. Specifically, we asked subjects (1) to guess the purpose of the experiment, (2) to guess the purpose of the gender discrimination task, (3) whether they perceived any relationship between the faces and the dice rolls, (4) if they thought the dice rolls were fair, (5) if they were aware they could cheat, and (6) if they actually had cheated. Participants then drew a number from 1 to 40 at random from a tin, and were paid the value of that trial (see below). All subjects also received an additional, unexpected, 2 SGD for participating in the experiment to raise the average expected payment to 5.50 SGD.

### Experimental paradigm

The priming paradigm in this experiment was similar to one we used previously to test the effects of disgust priming on judgments in moral dilemmas (Ong et al., 2014). During the task blocks, subjects completed two tasks in alternating order, a gender-discrimination task adapted from Winkielman et al. (2005), which was the priming phase, and a slightly modified dice-rolling task that was used in Jiang (2013) and Ariely et al. (2014) to assess levels of honesty (Figure 1). Stimuli were presented on Windows computers using E-Prime 2.0 (Schneider et al., 2002).

Details of the priming phase have been reported previously (Ong et al., 2014). Briefly, subjects were first presented with a forward and backward masked facial prime (16.66 ms for each), with either a disgust or neutral facial expression. The same person's neutral-expression face was then presented, and participants were asked to classify their gender. The disgust and neutral blocks each had an equal number of male and female faces, presented in a random sequence. Facial images were adapted from the Karolinska Directed Emotional Faces (KDEF) database (Lundqvist et al., 1998).

Following their response to the gender discrimination query, participants were prompted to choose between “top” or “bottom,” and to remember their selection. They then rolled a physical 6-sided die and were asked to write down the corresponding number on a piece of paper. For example, if they rolled a “5,” they were supposed to record “5” if they had chosen “top,” and “2” if they had chosen “bottom.” The six possible top-bottom combinations are presented in Figure 1. This procedure provides two different ways in which the participant can be dishonest: either by falsifying the side they had thought of (e.g., recording “5” when they had selected “top” and rolled a “2”), or by recording a number that had appeared on neither the top nor the bottom of the rolled die.

## Results

### Gender classification task

Accuracy on the gender classification task was high (93.5%, SD = 0.7%), indicating that subjects were attending to the stimuli during the gender discrimination component (and the subliminal face presentations). We excluded two subjects with low (<80%) accuracy from subsequent analysis; data from the remaining 48 subjects are reported from this point.

### Effect of coral-reef movie clip on mood

To minimize carry-over effects of priming from the first to the second block, participants viewed a documentary video on coral reefs. Although we did not collect subjective mood ratings in the current experiment, we did measure them in a previous study, in which the same video clip was used (Ong et al., 2014). Subjective disgust, anger, sadness, and happiness were measured at multiple time points in that experiment. To test whether the video returned participants to their baseline mood levels in our previous dataset, we conducted paired-sample *t*-tests between ratings on the first (pre-task) mood survey, and post-video mood levels, and found no significant differences (all differences *p* > 0.0125 after correction for multiple comparisons for the 4 moods). This indicates that the coral-reef video has the desired effect of returning mood to baseline levels.

### High likelihood of dishonest reporting

**Figure 2:**
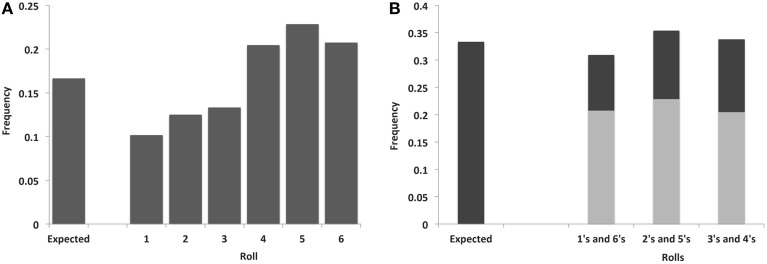
**Frequency distribution of reported rolls**. **(A)** by individual number and **(B)** combining across paired-opposite numbers. The expected frequency of rolls by chance is plotted on the left of each graph.

We first tested for evidence of dishonesty across our entire sample of participants, by examining whether the observed proportions of reported rolls of each number (1–6) concurred with the expected proportion for each number across all trials (16.7%) (Figure 2A). This revealed a highly significant deviation from the expected uniform proportions (*X*^2^ = 87.08, *df* = 5, *p* < 10^−37^; Cramer's *V* = 0.21), indicating an extremely high likelihood that some subjects were not honestly reporting the outcomes of their die rolls (percentages: 1 = 10.2%, 2 = 12.5%, 3 = 13.3%, 4 = 20.5%, 5 = 22.9%, 6 = 20.8%).

To confirm, we compared the mean reported dice roll values (MRDRV) for each participant, across all trials, against the expected mean of 3.5. This analysis revealed a significantly higher MRDRV than would be expected by chance [mean = 3.98, SD = 0.50, *t*_(47)_ = 6.65, *p* < 10^−7^, *d* = 0.96]. This indicates that, as a group, subjects were making dishonest reports by writing down numbers higher than those that would be expected by chance. Cheating behavior was also detectable separately in both the disgust and neutral blocks [disgust: mean = 3.91, SD = 0.59, *t*_(47)_ = 4.76; *p* < 10^−4^, *d* = 0.69; neutral: mean = 4.04, SD = 0.54, *t*_(47)_ = 6.82, *p* < 10^−7^, *d* = 0.99].

### Two types of dishonesty present

There are two different ways in which a participant can be dishonest in this paradigm: reporting the chosen side (top/bottom) dishonestly, or fabricating a value that is on neither the top nor the bottom of the die. The latter behavior can be detected by comparing whether there is a significant deviation from a uniform distribution when combining the paired opposite sides (1's + 6's, 2's + 5's and 3's + 4's). A chi-square test indicated that the observed distribution was significantly different from chance (*X*^2^ = 6.11, *df* = 2, *p* = 0.047; Cramer's *V* = 0.06), with post-hoc tests showing fewer 1's + 6's (*X*^2^ = 5.34, *df* = 1, *p* = 0.02; Cramer's *V* = 0.05) and marginally more 2's + 5's (*X*^2^ = 3.67, *df* = 1, *p* = 0.055; Cramer's *V* = 0.04) than expected (Figure 2B), indicating that our observed distributions cannot be accounted for based solely on participants performing within-pair replacements (e.g., substituting a 5 for a 2 or a 6 for a 1).

### Main effect of disgust priming on dishonesty

**Figure 3:**
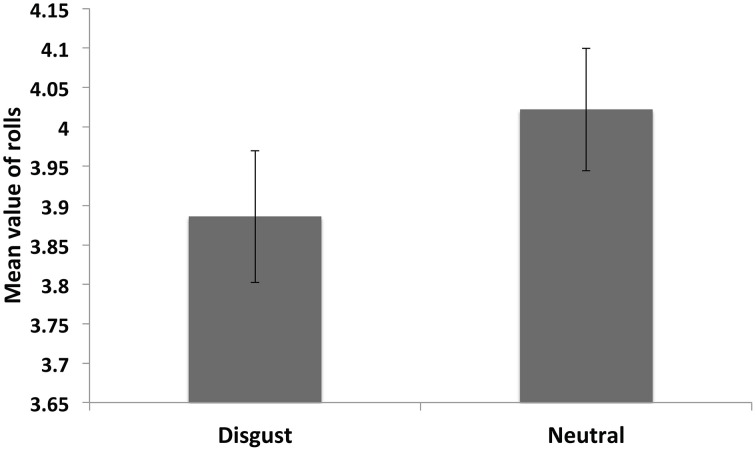
**Main effect of disgust presentation**. Subjects (*N* = 49) were slightly less dishonest in the disgust than the neutral condition, but this effect was not significant.

We tested the main effect of disgust facial expression priming by comparing the mean reported roll value between the disgust and neutral blocks (Figure 3: Neutral, mean = 4.04, SD = 0.54; Disgust, mean = 3.91, SD = 0.59). On average, the mean reported value was lower in the disgust priming condition (mean difference = −0.13, SD = 0.54), but this difference reached only marginal significance [Figure 3, *t*_(47)_ = −1.68, *p* = 0.10].

### Increased dishonesty in the second task block

We compared the MRDRV between the first and second blocks of the task, and found that over-reporting was significantly higher in the later block [*t*_(47)_ = −3.10, *p* = 0.003, *d* = 0.39]. This order effect resulted in an average increase in reported roll of 0.22 (1st block: mean = 3.86, SD = 0.60; 2nd block: mean = 4.08, SD = 0.52).

### Disgust priming modulates dishonest reporting based on individual sensitivity to disgust

**Figure 4:**
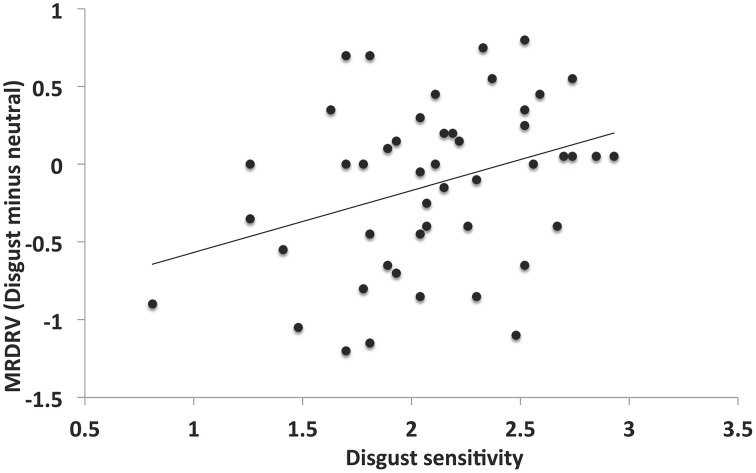
**Bi-directional relationship between disgust sensitivity and modulation of dishonesty**. The difference in the mean reported dice roll values between blocks was significantly positively correlated with disgust sensitivity, indicating replication of the bi-directional function of individual sensitivity determining the sign and size of moral modulation due to disgust stimuli.

Our principal aim was to test whether our bidirectional function (Ong et al., 2014; moral judgment modulation moderated by individual sensitivity) generalizes to the moral domain of honesty. To start, we tested the correlation between the difference in MRDRVs (Disgust—Neutral) and individual disgust sensitivity (DS) (measured by the DS-R scale). This revealed a significant positive relationship (Figure 4: *r* = 0.33, *p* = 0.02). We proceeded to conduct a One-Way repeated-measures ANCOVA using DS-R as a covariate to compare the difference in MRDRV between disgust and neutral blocks. This revealed a significant effect [*F*_(1, 46)_ = 7.38, *p* = 0.0009, η^2^_p_ = 0.14], suggesting the successful generalization of our bidirectional function to the moral domain of honesty.

Our initial analyses revealed that block order had a significant effect on MRDRV. This suggested that priming order (i.e., whether subjects performed the disgust or neutral block first) may also have had a significant effect on the difference in MRDRV between conditions (disgust minus neutral). A paired-sample *t*-test confirmed this difference [*t*_(47)_ = −3.09, *p* = 0.003; *d* = 0.89], with clear order-related differences (disgust first: mean difference = −0.34, SD = 0.49, neutral first: mean difference = 0.10, SD = 0.50). To confirm that our priming manipulation function was not produced as an artifact of this strong order effect, we ran a second ANCOVA including the priming order as a second covariate (in addition to DS-R). This model revealed a significant prime ^*^ order effect [*F*_(1, 45)_ = 9.92, *p* = 0.003, η^2^_p_ = 0.18], but also resulted in an increase in the main effect of prime [*F*_(1, 46)_ = 17.17, *p* = 0.0001, η^2^_p_ = 0.28].

We also examined whether there was an effect of manipulation order on the disgust sensitivity reported by participants. While this metric is considered trait-like, with high test-retest reliability, it is certainly possible that the order of the disgust modulation could result in alterations in reported disgust sensitivity. We did not find a significant effect of manipulation order on the disgust sensitivity scale [disgust first: mean = 2.08, SD = 0.36; neutral first: mean = 2.12, SD = 0.54; *t*_(46)_ = −0.29, *p* = 0.78].

### Replicating analyses using a probabilistic approach

**Figure 5:**
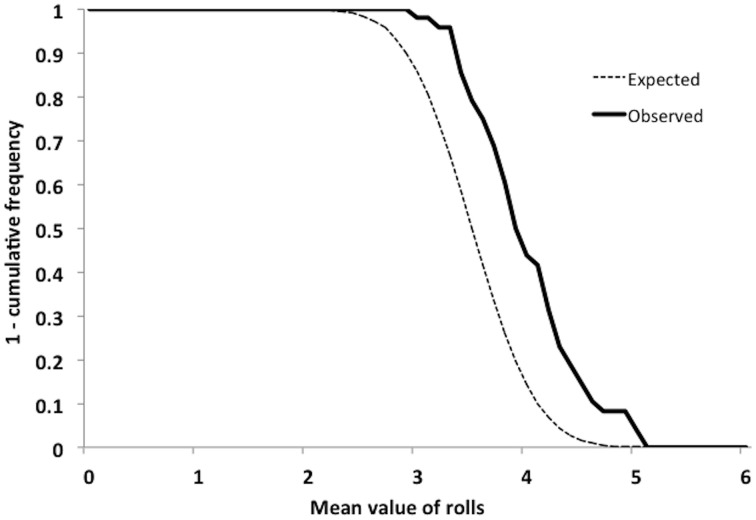
**Probability of honest reporting**. Expected and observed cumulative probabilities (subtracted from one) of obtaining mean values of 0–6 for 40 die rolls.

To confirm that these results were not artifactual, perhaps due to the MRDRV capturing both the variance associated with dishonest reporting as well as randomness attributable to the dice rolls themselves, we repeated the analyses using a probabilistic approach. This was accomplished by calculating the likelihood that the MRDRV reported by a subject was obtained by chance, based on a cumulative distribution function (CDF). These CDFs were converted to probabilities of honest reporting, by subtracting the CDF value from 1 (Figure 5). A feature of this probabilistic model is that it takes into account the directionality of signed deviations from the expected mean MRDRV of 3.5—it assumes that MRDRVs decreasing from 3.5 are increasingly likely honest reports, while values increasing from 3.5 are increasingly likely to be dishonest.

Repeating our analyses using the probability of honest reporting as a dependent variable yielded highly similar results to those using the MRDRV. The difference in probabilities (Disgust—Neutral) was correlated with DS-R (*r* = 0.29, *p* = 0.048). One-Way repeated-measures ANCOVA with DS-R and priming order included as covariates again revealed a significant effect of prime [*F*_(1, 46)_ = 11.94, *p* = 0.001, η^2^_p_ = 0.21] and a significant prime ^*^ order effect [*F*_(1, 45)_ = 7.16, *p* = 0.01, η^2^_p_ = 0.14].

### Common bidirectional function across moral domains of harm and honesty

**Figure 6:**
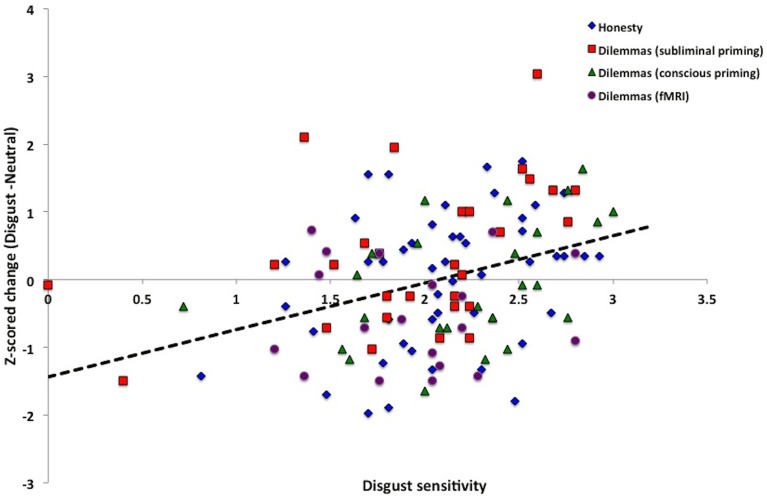
**Common bidirectional function**. Data from four independent experimental samples (*N* = 125), testing the effects of disgust presentation on honesty and judgment of moral dilemmas. Combined, these data obeyed the same bidirectional function, without significant main effect or interaction with task domain.

The above results indicate that the disgust priming effect on judgments of moral dilemmas generalizes to the moral domain of honesty. We next sought to test if all these data obeyed the same bidirectional function. To achieve this, we standardized the behavioral measures across experiments by converting the dependent variable in this study (MVRDR, *N* = 48), as well as our changes in moral acceptability variables from our previous behavioral studies (*N* = 54; Ong et al., 2014), and fMRI experiment (*N* = 19; Lim et al., unpublished), to z-scores. We then correlated this entire set of scores with DS-R. This overall correlation was significant (Figure 6: total *N* = 121, *r* = .34, *p* = 0.0002). Furthermore, One-Way ANCOVA of the z-scored data with DS-R as a covariate revealed a significant effect of priming across the four different datasets [*F*_(1,119)_ = 13.44, *p* = 0.0003, η^2^_p_ = 0.10].

To test whether data across the two moral domains obey the same function, we performed a linear regression using z-scored change (disgust minus neutral) as a dependent variable, and DS-R, study type (dilemmas and honesty), and an interaction term (study type ^*^ DS-R) as predictors. The main effect of moral domain was not significant (β = −0.08, *t* = −0.19, *p* = 0.85), nor was its interaction with DS-R (β = 0.08,*t* = 0.19, *p* = 0.85), indicating that the slopes of data from the two task paradigms did not differ significantly.

### Relation of dishonesty to luck perception and religiosity

Self-reported luck perception was weakly associated with MRDRV (*r* = −0.26, *p* = 0.08); subjects who perceived themselves as being luckier tended to have more honest reporting. There was no significant association between religiosity and MRDRV (*r* = −0.006, *p* = n.s.).

### Cheating and lying? few subjects admit to dishonesty in post-task debriefing

In the post-experiment debriefing, subjects were asked whether they were aware they could cheat during the experiment, and whether or not they actually cheated. 34 out of 48 (70.8%) of participants indicated that they were aware they could be dishonest, while only 6 out of 48 (12.5%) admitted to dishonest behavior. Those denying awareness that they could cheat actually had slightly higher MRDRVs (indicators: mean = 4.03, SD = 0.15; non-indicators, mean = 3.95, SD = 0.83), but this difference did not reach significance [*t*_(46)_ = −0.50, *p* = 0.61].

None of the subjects guessed the purpose of the experiment or reported detecting the facial primes. None indicated that they thought that the die was unfair, or that the faces and die rolls were connected in any way.

## Discussion

In this study, we examined whether the effects of disgust stimuli on moral judgments of hypothetical personal harm would generalize to the domain of dishonesty (Ong et al., 2014). We find that they do. Moreover, we find not only the presence of an effect, but that it is indistinguishable from the previously specified effect—a bi-directional function with individual DS moderating the direction and size of the effect. This suggests that the same cognitive and neural mechanisms may underlie the impact of disgust-expression stimuli on both harm judgments and honesty decisions. In addition, by utilizing an incentive-compatible economic task, we show that these effects are not limited to judgments about third parties in hypothetical situations, but generalize to outcomes with immediate real-world effects on the participants.

### Disgust alters moral decisions

Numerous experiments have shown that disgust stimuli alter moral judgments (Chapman and Anderson, 2014). Early studies reported that a variety of methods such as the use of dirty environments (Schnall et al., 2008b) and hypnotic suggestion of disgust (Wheatley and Haidt, 2005) result in more severe judgments of moral transgressions. However, other researchers have recently reported effects in the reverse direction (Cummins and Cummins, 2012; La Rosa et al., 2012; Olatunji et al., 2012). These disparate results could be reconciled by our recent discovery that DS moderates the effect of both subliminal and consciously presented disgust stimuli on moral judgments (Ong et al., 2014). Disgust stimuli result in more utilitarian judgments in individuals with high DS, and more deontological judgments in those low in DS.

Far fewer studies have examined the effects of disgust stimuli on moral decisions with real-world or incentive-compatible outcomes. Two such studies utilized the ultimatum game to examine this issue, and found opposite directions of effect. Moretti and di Pellegrino (2010) found that disgust induction using IAPS pictures caused subjects to reject unfair offers more frequently than those primed with sad or neutral images, and Bonini et al. (2011) showed the opposite effect, using environmental disgust smells as their disgust stimuli. Neither of these studies reported measures of disgust sensitivity, and it is possible that their effects are driven by the same bi-directional function we find, with differences in mean sample disgust sensitivities driving their differential mean shifts. We elaborate on this possibility in the following section.

### Concurrent bi-directional effects of disgust stimuli on harm and honesty judgments

An important conclusion of our previous work was that main effects of disgust priming on moral decisions might not be detectable without measuring disgust sensitivity as a moderator (Ong et al., 2014). Indeed, in the current study, the main effect of priming was only marginally significant (*p* = 0.10), and it was only by covarying individual sensitivity that the manipulation effect reached significance. This finding accords with other work showing that disgust sensitivity moderates attitudes toward perceived moral transgressions and violations of social norms (Inbar et al., 2009; Chapman and Anderson, 2014).

In our prior behavioral experiment (Ong et al., 2014), we found that disgust stimuli led high sensitivity participants to find utilitarian actions more acceptable. We find the same pattern of effect in the current experiment. In the die-rolling task, disgust stimuli lead high sensitivity participants to greater reported MRDRVs—indicating that they are enhancing the reported die roll, which is the maximizing action as it increases their likely payoff. Low sensitivity individuals in each experiment showed increased deontological judgments—lower stated acceptability of utilitarian harm actions in Ong et al. (2014), and, in the current, more honest MRDRV reporting.

Interestingly, while these functions match very well, the maximizing behavior of cheating is not a utilitarian action—it is maximizing self-oriented payout that does not maximize the common good. In other words, the disgust primes are not simply driving behavior toward/away from utilitarian/deontological actions. Rather, there must be an underlying informational factor that is being modulated and causing these behavioral changes. As examples, such behavioral changes may be the result of modulating the inclusion of information about the consequences of the actions, such as possible punishments (for killing one to save many or cheating) or rewards (esteem for saving lives or monetary). This would suggest that the relative value of the options is altering, driven either by increases in the subjective value of rewards, or decreases in the subjective value of punishments (Becker, 1968). For example, we could speculate that the disgust primes are altering the degree of concern for social blame, which if reduced would result in a willingness to kill one person to save others (vignettes) and a greater likelihood to cheat in the die-rolling task.

In summary, our data show that disgust stimuli alter moral decisions beyond hypothetical scenarios to those with real world outcomes. This is important, as moral dilemmas such as the ones in our previous experiments have been criticized for having a large number of uncontrolled variables (Christensen and Gomila, 2012) and may lack external validity (Bauman et al., 2014).

### Multiple effects, one mechanism?

We found evidence that a single bidirectional function may govern the results of our experiments across moral domains, by z-scoring and combining our data across this experiment and our previous studies on harm judgments. When standardized, the behavioral change data across studies combined to a significant bi-directional function, with no significant main effects or interaction of moral domain. The common function shows that disgust stimuli are modulating moral judgments in an indistinguishable manner across these two disparate tasks (a bidirectional function dependent on individual sensitivity). Intriguingly, this suggests that the DS-R scale may be capturing a core individual difference that is responsible for modulating moral behavior in the presence of disgust across moral domains. It also suggests the same brain regions may underlie this translation, with the temporal-parietal junction being a prime candidate based on our previous research (Lim et al., unpublished). This is in spite of the fact that different neural networks appear to be responsible for processing the unique facets of harm and dishonesty judgments (Parkinson et al., 2011), suggesting that our effects operate late in the moral decision-making process. Furthermore, it is unclear how specific this effect is, that is, whether it is the result of disgust induction or a general arousal effect that would be present across emotional domains. We specifically note that our prior study indicated that the subliminal presentation of disgust facial expressions did not result in a significant induction of disgust feelings in our participants (Ong et al., 2014).

### Cheating is more prevalent in the latter half of the task

Replicating the findings of Jiang (2013), we found that MRDRV was significantly higher in the second than the first block. We also found a significant interaction effect between the main effect of priming and priming order. These results highlight the importance of counterbalancing and statistical control of priming order in replications of this work.

### Order effect—moral inertia

We found a significant behavioral order effect that interacted with our manipulation effect. If a participant first experienced the neutral condition and then the disgust condition, they were more likely to cheat in the disgust condition. However, if a participant first experienced the disgust block and then the neutral block, they were likely to begin cheating in the disgust block and then continue to do so through the neutral block. We were able to significantly identify the bi-directional function without inclusion of the order effect, but the function strengthened by accounting for the order effect as well.

Previous studies have also identified order effects in moral decision making, related to the order of stimuli in both hypothetical (Schwitzgebel and Cushman, 2012) and real outcomes (Gold et al., 2015). In our initial experiments that uncovered the bi-directional moderation of disgust sensitivity through which disgust facial expression alter moral judgment (Ong et al., 2014), we were careful to control for the order of the hypothetical vignettes across participants. The current results clearly show the impact of recent decisions on behavior, with our participants demonstrating a form of moral inertia—maintaining the modulation of moral acceptability for the remainder of the experiment, even with the videos intended to wash out the manipulation effects.

## Conclusion

Subliminal disgust stimuli alter cheating behavior in an incentive-compatible task based on individual sensitivity to disgust. This demonstrates the generalizability of this effect across multiple moral domains and that it extends from hypothetical dilemmas to real-world outcomes. These unconscious inputs interact with individual differences in personality to modulate moral judgments of both harm and dishonesty.

Disgust is just one example of a stimulus type that can influence real-world moral decision making. Discovering and reporting the general features of stimuli that affect moral cognition is an important future endeavor with the potential to alter the way individuals think about the way they make moral choices. As even small transgressions can have tremendous costs in aggregate, the impact of understanding moral cognition could play a profound role in decision-making in future human society.

## Author contributions

JL and OM developed the study concept and design. Data collection was performed by PH under the supervision of JL and OM. Data analysis was performed by JL and PH under the supervision of OM. All authors contributed to drafting and revising the manuscript, and all authors approved the final version of the manuscript for submission.

### Conflict of interest statement

The authors declare that the research was conducted in the absence of any commercial or financial relationships that could be construed as a potential conflict of interest.
